# The State of the Nitric Oxide Cycle in Respiratory Tract Diseases

**DOI:** 10.1155/2020/4859260

**Published:** 2020-10-19

**Authors:** Svetlana Soodaeva, Igor Klimanov, Nailya Kubysheva, Nataliia Popova, Ildar Batyrshin

**Affiliations:** ^1^Pulmonology Scientific Research Institute under FMBA of Russia, Orekhovyy Bul'var 28, Moscow 115682, Russia; ^2^Kazan Federal University, 18 Kremlyovskaya St., Kazan 420000, Russia; ^3^Centro de Investigación en Computación, Instituto Politécnico Nacional (CIC-IPN), Av. Juan de Dios Bátiz, Esq. Miguel Othón de Mendizábal S/N, Gustavo A. Madero, 07738 Mexico City, Mexico

## Abstract

This review describes the unique links of the functioning of the nitric oxide cycle in the respiratory tract in normal and pathological conditions. The concept of a nitric oxide cycle has been expanded to include the NO-synthase and NO-synthase-independent component of its synthesis and the accompanying redox cascades in varying degrees of reversible reactions. The role of non-NO-synthase cycle components has been shown. Detailed characteristics of substrates for the synthesis of nitric oxide (NO) in the human body, which can be nitrogen oxides, nitrite and nitrate anions, and organic nitrates, as well as nitrates and nitrites of food products, are given. The importance of the human microbiota in the nitric oxide cycle has been shown. The role of significant components of nitrite and nitrate reductase systems in the nitric oxide cycle and the mechanisms of their activation and deactivation (participation of enzymes, cofactors, homeostatic indicators, etc.) under various conditions have been determined. Consideration of these factors allows for a detailed understanding of the mechanisms underlying pathological conditions of the respiratory system and the targeting of therapeutic agents. The complexity of the NO cycle with multidirectional cascades could be best understood using dynamic modeling.

## 1. Introduction

Nitric oxide (NO), until recently, was known mainly as a toxic gas in the atmosphere of large cities and was considered primarily, even almost totally, from the point of view of environmental pollution [1].

However, a large number of studies in the field of vascular physiology, pathophysiology, neurology, biochemistry, pharmacology, and immunology have convincingly shown that this molecule is synthesized in living organisms and has a broad spectrum of bioregulatory action [2–4].

In 1991, Gustafsson et al. found nitric oxide in exhaled air in animals and healthy people [5], and further changes in NO level in exhaled air were detected in a number of respiratory system diseases (bronchial asthma, bronchiectasis, systemic connective tissue diseases, sleep apnea syndrome, pulmonary tuberculosis, complications after lung transplantation, and cystic fibrosis) [2, 3, 6]. The evidence based on the contribution of NO to the pathogenesis of many respiratory tract diseases has been accumulated [7].

**Scheme 1:**
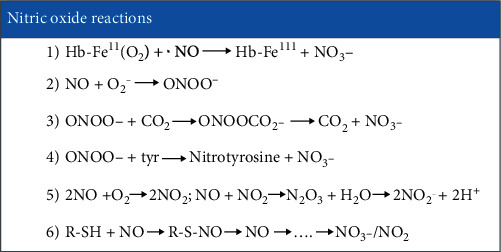
O_2_ complex with hemoglobin (Hb-O_2_) and other heme proteins oxidize it to nitrate anion (NO_3_^−^) (1); when interacting with superoxide (О_2_^−^), toxic peroxynitrite (OONO^−^) is formed (2). In the absence of other reactive molecules, they are combined to form NO_3_^−^ and CO_2_. But in the presence of reacting molecules, for example, tyrosine, NO_3_^−^ interacts with the resulting tyrosyl radical (tyr + CO_2_^−^⟶tyr۰+CO_2_) with the formation of nitrotyrosine (3 and 4). Small amounts of NO are reduced to N_2_O. NO is oxidized to NO_2_ dioxide, which gives higher oxides of nitrogen (N_2_O_3_ and N_2_O_4_) and many other reactions [8] (5). Thus, nitric oxide is able to interact with thiol groups (R-SH) and with iron-containing complexes, which play a leading role in the transportation and deposition of NO (6) [9].

Currently, it is believed that NO is formed from arginine by NO synthase and enters into many competing reactions that are presented in Scheme 1.

### 1.1. NO in Respiratory Tract Pathologies

Exhaled nitric oxide (FeNO) is a sensitive, reproducible, and noninvasive marker of eosinophilic airway inflammation. Accordingly, FeNO is widely used to diagnose and monitor the effectiveness of therapy in the treatment of asthma. In patients with asthma, the high content of FeNO which decreased in response to treatment by corticosteroids was revealed. An increase in the FeNO content in mild and moderate bronchial asthma (BA) during exacerbation in comparison with the control and a group of patients with severe BA has been shown. In patients in the latter group, the concentration of FeNO was at the level of control values. Currently, it is associated with a direct inhibitory effect of oral and inhaled glucocorticosteroids on NOS, as well as changes in the level of cytokines and a modification of the inflammatory response [10–12].

FeNO measurement has been shown to be an alternative diagnostic tool compared to conventional lung function tests for the diagnosis of bronchial asthma [10, 11]. Analysis of NO metabolites revealed that the concentration of nitrotyrosine in exhaled breath condensate (EBC) increases with mild asthma. In the same study, its level was reduced in moderate and severe BA compared to the control group. The nitrotyrosine content correlated with the level of FeNO only in mild BA. At the same time, the study of another nitric oxide metabolite, nitrosothiol (RS-NO), showed an increase in its concentration with moderate asthma compared with the control group and patients with mild disease severity [13].

Most patients with chronic obstructive pulmonary disease (COPD) did not show a high level of FeNO. However, in some patients with COPD, elevated FeNO values have been reported. It has been shown that this may be associated with asthma-COPD overlap [14].

In COPD, an increase in the content of nitrites and nitrosothiols in EBC has also been shown [13, 15, 16]. But, in general, data relating to NO and its metabolites in EBC (nitrates, nitrites, and 3-nitrotyrosine) in COPD remain contradictory. Some authors have shown the relationship between NO and its metabolites, as well as the severity of COPD. At the same time, other researchers did not find significant associations of NO level with its derivatives [17–19].

In patients with cystic fibrosis (CF) in remission, an increase in the content of nitrite anion in EBC was revealed, in contrast to the concentration of FeNO, the value of which was within the normal range. Increased levels of nitrate anion and nitrotyrosine in the sputum in patients with CF with normal levels of NO have also been shown [20, 21].

Anil et al. studied nitrite levels in induced sputum as a noninvasive marker for assessing the degree of inflammation in children with CF in remission [22]. Also, in this work, the relationships between nitrite levels in induced sputum and lung function have been analyzed. As a result, an increase (more than three times) in the level of nitrites in the induced sputum was revealed compared with the control [22]. A positive correlation between the number of neutrophils and leukocytes and the level of this metabolite of nitric oxide has been observed. In addition, a negative correlation was also found between the concentration of nitrites and FEV_1_ (forced expiratory volume in one second). Based on the data obtained, the authors suggest that a significant amount of NO is stored in the respiratory tract fluids in the form of metabolites (nitrites). According to the authors, these nitrite levels indicate the degree of inflammation in the airways with CF and not the concentration of FeNO [22].

Grasemann et al. conducted a comparative study of nitric oxide metabolite levels in saliva and sputum in patients with CF [23]. As a result, an increase in the salivary concentration of nitrate and nitrite anions was established both with a stable course and with an exacerbation of this disease compared with the control group. It was also noted that the level of NO metabolites in saliva was higher than in sputum. Moreover, the level of FeNO in patients with CF was lower compared with the control group. The authors suggest that a decrease in the concentration of NO in exhaled air in patients with CF may be due to the peculiarities of NO metabolism in airway secretions [23].

Interesting data were obtained by studying the possibility of using indicators of FeNO and inflammatory markers (IM) in EBC (pH, nitrites, nitrates, hydrogen peroxide (H_2_O_2_), 8-isoprostane, and Th1/Th2 cytokines) to detect (exacerbate) CF and by studying the ability of these noninvasive IMs to indicate the CF severity [24]. It was found that in CF, the concentration of interferon (IFN-c) and nitrite in EBC was significantly higher, while the levels of FeNO were lower compared to the control. When using multivariate logistic regression models, the presence of CF was best indicated by 8-isoprostane, nitrite, and IFN-c. Exacerbation of CF was best indicated by 8-isoprostane and nitrite. The most significant biomarkers of CF severity were FeNO and pH of the condensate. Thus, the authors believe that this combination of different exhaled IMs can indicate the presence (exacerbation) of CF and the severity of the disease in children [24].

Based on our experience in studying NO metabolism in various diseases of the respiratory tract, it seems promising to search and study the most significant metabolites of nitric oxide and their relationships with each other and with other disease markers. These studies may turn out to be the most diagnostically valuable approach in understanding the pathogenetic mechanisms of the development of respiratory pathologies, including CF.

Despite the fact that to date, there is a huge amount of evidence on the generation of NO and its metabolites in various respiratory pathologies, the exact contribution of NO and/or its metabolites to the inflammatory diseases of the lungs is still unclear. Indeed, NO can play completely different roles during the stages of inflammatory respiratory disease development. In acute and severe conditions, its proinflammatory effect is possible, and in more stable conditions, NO can have an anti-inflammatory effect [25].

**Figure 1:**
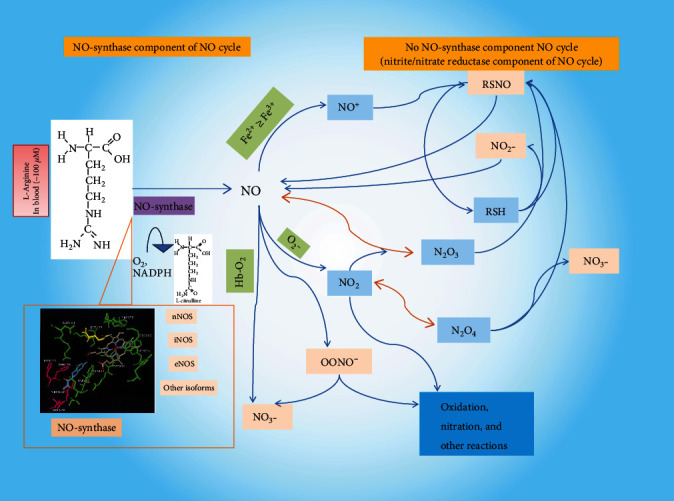
Nitric oxide (NO) cycle. NO_2_^−^: nitrite anion; NO_3_^−^: nitrate anion; NO, N_2_O_3_, NO_2_, and N_2_O_4_: nitric oxides I, II, III, and IV, respectively; RSH: thiols; RSNO: nitrosothiols; NO^+^: nitrosonium; OONO^−^: peroxynitrite; O_2_^−^: superoxide anion radical.

Currently, attempts to search for diagnostically significant correlations of NO and its metabolites with various respiratory pathologies and the degree of their manifestation remain insufficiently effective. Therefore, we have proposed an extension of the concept of a nitric oxide cycle. It includes the NO-synthase and NO-synthase-independent component of its synthesis and the accompanying redox cascade, in varying degrees of reversible reactions [26–28] (Figure 1).

In the vast majority of in vivo and in vitro studies of recent decades, the modification of NO production and the following cascade of molecular and functional reactions are considered in the context of changes in the generation of NO in the NO synthase component of the nitric oxide cycle [2–4, 6, 7, 11, 12]. The functioning NO-synthase-independent component of the cycle and the identification of the molecular mechanisms of its components and the system as a whole is of special interest.

### 1.2. The Pathways of Nonenzymatic Synthesis of NO from NO-Synthase-Independent Component of the Nitric Oxide Cycle

Despite the fact that chemical reactions accompanied by the release of nitrogen oxides were studied in inorganic chemistry for a long time, their place in physiological processes, before the discovery of endogenous NO synthesis, seemed impossible.

The following nitrogen oxides are known: nitrogen oxide (I) N_2_O, nitrogen oxide (II) NO, nitrogen oxide (III) N_2_O_3_, nitrogen oxide (IV) NO_2_, and nitrogen oxide (V) N_2_O_5_, in which oxidation states from +1 to +5 are exhibited. Oxides NO, NO_2_, N_2_O_4_, and N_2_O_3_ quite easily turn into each other, and these transformations are considered as nonenzymatic reactions of nitric oxide. Almost all of these reactions are reversible reactions [8]. The forward and reverse reactions have different rate constants (*k*), and, depending on the microenvironment conditions, concentrations of substrates and products differ [8].

**Figure 2:**
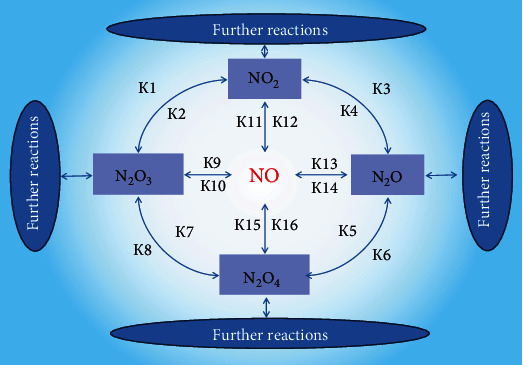
Intertransformations of higher nitrogen oxides. N_2_O, NO, N_2_O_3_, NO_2_, and N_2_O_4_: nitric oxides I, II, III, IV, and V, respectively; *k* (1-16): reaction rate constants.

In this regard, it is possible to regulate the concentration of nitric oxide in specific local conditions in vivo. The dissolution coefficients of NO, in particular, are different for the hydrophobic and hydrophilic phases. Therefore, the phase component of the regulation of these reactions is of particular importance (Figure 2).

The following reactions are known for producing NO. For example, nitric oxide is formed in the reaction of HNO_3_ interaction with some metals [29], in particular, with copper, which is included in the active centers of a number of enzyme systems:

(1) $$\begin{array}{c} 3\text{Cu}+8\text{HN}{\text{O}}_{3}⟶3\text{Cu}\left(\text{N}{\text{O}}_{3}\right)2+2\text{NO}+4{\text{H}}_{2}\text{O} \end{array}$$

The source of nitric oxide are other nonenzymatic reactions:

(2) $$\begin{array}{c} \text{FeC}{\text{l}}_{2}+\text{NaN}{\text{O}}_{2}+2\text{HCl}⟶\text{FeC}{\text{l}}_{3}+\text{NaCl}+\text{NO}+{\text{H}}_{2}\text{O}\\ 2\text{HN}{\text{O}}_{2}+2\text{HI}⟶2\text{NO}+{\text{I}}_{2}+2{\text{H}}_{2}\text{O} \end{array}$$

These reactions occurring in vivo are usually not considered. However, a number of authors registered the release of NO in the presence of hydrochloric acid, in particular, when the nitrite of saliva hits the stomach [30, 31].

There is a small amount of information collected from the study of the role and changes in the content of other nitrogen oxides in patients with lung diseases.

In particular, the effect on NO_2_ of the lungs as an air pollutant, which causes the formation of free radicals, lipid peroxidation, oxidative damage to proteins, increased proliferation, production of proteases by macrophages, etc., has been explored [32]. It is also shown that the short-term effect of NO_2_ has a negative impact on the parameters of lung function, and it increases the concentration of isoprostane-8 in EBC among students without respiratory pathologies. A direct correlation was found between the concentration of NO_2_ and the content of isoprostane-8 and the inverse association between FEV_1_ (forced expiratory volume in one second) and NO_2_ [33]. The elevated NO_2_ concentrations in the air have been shown to increase the frequency of asthma exacerbations and rhinoconjunctivitis in the urban population [34, 35].

### 1.3. Organic Nitrates as a Substrate for the Synthesis of NO in the NOS-Independent Component of the Nitric Oxide Cycle

A number of substances which increase NO availability have been applied successfully as medicine. Nitroglycerine (NG) (1,2,3-trinitroksipropan), being an ester of glycerin and nitric acid and a pharmacological medicine from the group of organic nitrates, has been used for over 100 years. The main property of these drugs is the ability to cause relaxation of vascular smooth muscle. Organic nitrates, as well as sydnonimines (molsidomine), are nitrovasodilators. Organic nitrates (nitroglycerin, isosorbide dinitrate, and isosorbide mononitrate) are the most common in clinical practice [36]. The effects of these compounds, mediated by the functioning of nitrate/nitrite reductase components, correspond to the proposed concept of the nitric oxide cycle. It is believed that the main active compound in the application of organic nitrates is NO or S-nitrosothiols, followed by activation of guanylate cyclase (GC). It is through the reaction with GC that the role of NO as a signaling molecule is realized. The activation of GC leads to the transformation of the Mg+2-2-GMP complex into cGMP and subsequent activation of cGMP-dependent protein kinases, which ends by the regulation of phosphodiesterase and ion channel activity [37].

However, the molecular process of the biotransformation of NG to NO has not yet been studied. Many works consider only the effects associated with an already formed NO. It has been shown that the biotransformation products in NG tissues are 1,2-glycerol dinitrate, 1,3-glycerol dinitrate, nitrite anion, and NO or S-nitrosothiols. A number of intracellular enzymes are considered as the alleged participants in the NG reduction reaction, namely, glutathione-S-transferase, cytochrome P450 reductase, cytochrome P450, and xanthine oxidase [38]. The role of mitochondrial aldehyde dehydrogenase, as a participant in NG biotransformation, was also noted [38]. In addition, it was shown that under conditions of acute hypoxia, the release of NO from organic nitrates increases, clarifying their selective effect in ischemic foci [39].

The influence of the use of organic nitrites on the NO cycle in inflammatory lung diseases has not been studied. The effect of NO donors on pulmonary arterial hypertension has been shown [40, 41]. Intravenous use of 1,2-propanediol on a model of pulmonary embolism in rabbits has been found to increase the concentration of FeNO, unlike inorganic nitrite, and prevent lung hypertension [42]. A dose-dependent increase in FeNO in healthy lambs after intravenous administration of a trinitroglycerin or a nitroprusside has been noted [43].

### 1.4. Microbiota as a Participant in the Nitric Oxide Cycle

Natural microbiota can have a significant effect on the metabolism of many substances and processes in the host organism [44]. The transformation, in particular, of the respiratory microbiota with the consolidation of representatives of conditionally pathogenic and pathogenic flora is associated with exacerbations and severity of many chronic diseases of the respiratory tract [45, 46].

**Scheme 2:**
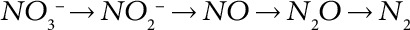
Reduction of nitrates and nitrites in prokaryotes.

It is known that a number of bacteria are able to release gaseous nitrogen oxides. Electron acceptors with high redox potential, such as nitrite and nitrate anions, are often used as reaction substrates. The reduction of nitrate to gaseous nitrogen oxides, the so-called nitrate respiration or denitrification, is a process of anaerobic respiration of bacteria. A series of enzymes is involved in the chain of reactions shown in Scheme 2: nitrate reductase, NO-forming nitrite reductase, nitric oxide reductase, and nitrous oxide reductase, respectively [45–47].

Denitrifying bacteria are widespread. The ability to denitrify was found in representatives of more than 40 genera of eubacteria and only in one group of archaebacteria. Among eubacteria, gram-negative proteobacteria of the genera *Pseudomonas*, *Alcaligenes*, *Paracoccus*, *Hyphomicrobium*, and *Thauera*, as well as some representatives of gram-positive bacteria of the genus *Bacillus*, have the ability to denitrify [47].

Theoretically, these pathways for the formation of nitrite and NO are also relevant in the human body. However, this issue remains virtually unexplored. Before the discovery of the endogenous synthesis of NO from arginine, the presence of nitrite in the human body was associated exclusively with the activity of the microflora of the gastrointestinal tract, although the presence of nitroso compounds in human tissues and fluids was known for a very long time [8]. A number of studies have been shown that oral microflora can reduce nitrates to nitrites and, lastly, to NO with subsequent regulation of blood pressure [48, 49].

Dietary and metabolic nitrates enter saliva from the blood through their active accumulation in the salivary glands and are reduced to nitrite and NO in the oral cavity by the action of local microbiota. It has also been shown that when consuming foods rich in nitrates, the formation of NO in the oral cavity leads to an increase in FeNO. The authors suggest that such data may be misinterpreted as an enhancement in inflammatory activity in the respiratory tract [50].

Also, the avalanche-like growth of NO metabolites in the blood during septic shock may be related to the degree of bacteriuria and/or the type of microflora [51]. Accordingly, the microbiota of the skin, gastrointestinal and respiratory tracts, etc., as well as their quantitative and qualitative changes in various physiological/pathophysiological processes, affect the nitric oxide cycle. More and more studies are devoted to studying the composition of the microbiota of the respiratory tract in various lung diseases [52–55].

Some pathogens, including *P. aeruginosa*, have a genetically determined ability to denitrify. The ability of microorganisms to use a wide range of electron acceptors to generate ATP provides them with metabolic flexibility in transitional environments, as these organisms live in different habitats. Kolpen et al. demonstrated the relationship between nitric oxide metabolites and the respiratory tract microbiota [56, 57] In particular, it was demonstrated that in cystic fibrosis in the anaerobic zones of endobronchial mucus, denitrification processes occur. The level of N_2_O was used as a marker of denitrification. The significant generation of N_2_O was found in the sputum of patients with CF with chronic *P. aeruginosa* infection. In this process, there was a decrease in initially high levels of NO_3_^−^ and NO_2_^−^ in sputum. According to the authors, these data indicate that denitrification can serve as an alternative metabolic pathway that allows *P. aeruginosa* to successfully develop in airway microniches with a lack of oxygen in CF patients [56].

It has been shown that the ability of microorganisms to perform denitrification correlates with their pathogenicity in CF. In the study of infectious isolates from 32 patients with CF, it was found that all the studied pathogens (*P. aeruginosa*, *Achromobacter xylosoxidans*, *Burkholderia multivorans*, and *Stenotrophomonas maltophilia*) grow under anaerobic conditions with the consumption of NO_3_^−^. However, denitrification recorded for N_2_O production was detected for *P. aeruginosa*, *Achromobacter xylosoxidans*, and *Burkholderia multivorans*, but was not found in *S. maltophilia* isolates. The ability to conduct denitrification may contribute to the pathogenicity of infectious isolates since complete denitrification promotes the most rapid anaerobic growth. The inability of *S. maltophilia* to multiply during denitrification and, therefore, to grow in an anaerobic environment with CF may explain its low pathogenicity in these patients [57].

Further study of the possible relationships between the composition of the microbiota and the components of the nitric oxide cycle in lung diseases seems promising for understanding the mechanisms of pathogenesis, as well as targeted treatment and prevention of respiratory diseases.

### 1.5. Inorganic Nitrates and Nitrites as a Substrate for the Synthesis of NO in the NOS-Independent Component of the Nitric Oxide Cycle

Nitrate and nitrite anions have been considered by a number of researchers as stable metabolites of nitric oxide and are present in living organisms in micromolar amounts [58]. In addition, a different, but a significant amount of these ions comes from food and water. Poisoning with an excessive content of nitrates and nitrites proves their active participation in metabolic processes in a living organism, by embedding in the links of the nitric oxide cycle [59].

It is known that eukaryotes, in particular, fungi and liver cells of animals are capable of releasing N_2_O, especially in an environment with nitrites. Unlike bacteria, as described above, this process, however, is not associated with obtaining energy and is carried out to detoxify the body from nitrites [46]. In plants, the assimilative reduction of nitrate to nitrite is used in biosynthesis reactions [60]. In humans, these processes are poorly understood. Some studies have been shown that nitrates and nitrites can be substrates for the synthesis of NO. The generation of nitric oxide by the skin when applying the nitrite solution to it was noted [61]. Matsunaga and Furchgott demonstrated the myorelaxation of an isolated rabbit aorta in a medium with sodium nitrite [62].

**Figure 3:**
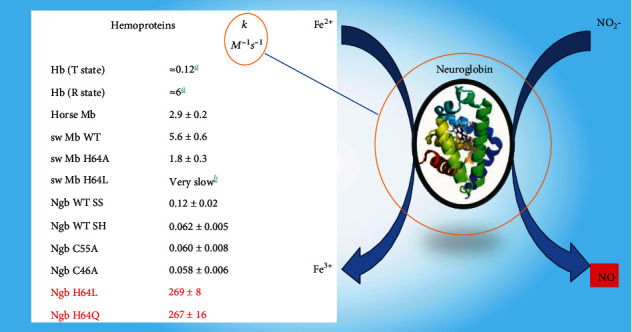
The action of globin proteins in the human nitrite reductase system. The figure shows the formation rate constants (*k*) of nitric oxide for various hemoproteins (Hb, Mb, and Ngb) of their nitrite anion. Neuroglobin has the highest rate of conversion of nitrite to nitric oxide (marked in red).

The main applicants for nitrite and nitrate reductase activity are the same groups of enzymes that are considered as in NG biotransformation. It has been shown that heme-containing proteins are capable of converting nitrites to NO with various rate constants (Figure 3). Among the currently studied hemoproteins, neuroglobin has the highest rate of conversion of nitrite to nitric oxide (Figure 3) [63, 64].

Neuroglobin is one of the vertebrate globin proteins involved in maintaining the gas homeostasis of the cell. It is an intracellular hemoprotein expressed in the central and peripheral nervous system, cerebrospinal fluid, retina, and endocrine tissues [64].

It is shown that hemoproteins are involved in NO_2_ reduction under hypoxic conditions at the site of protein expression similar to bacterial nitrite reductases. Poisoning by nitrates and nitrites is manifested primarily in methemoglobinemia with associated clinical symptoms, which is associated with the oxidation of heme iron under the action of excess nitrates/nitrites [64].

In our work studying the content of metabolites of the nitric oxide cycle in various diseases of the respiratory organs and comorbid conditions, it has been shown that among NO metabolites, nitrate anions have the highest concentration [65, 66]. The levels of nitrite anion vary from undetectable values (especially in EBC) to 20% of the total concentration of nitrate and nitrite anions. The ratio of nitrate to nitrite anions varies depending on the pathophysiological conditions [65, 66]. The concentrations also vary significantly depending on the biological environment under study, from units of *μ*M in EBC to hundreds of mM in urine [67].

We have studied the relationship of the components of the nitric oxide cycle in Chernobyl clean-up workers with COPD during antioxidant therapy with N-acetylcysteine (NAC) [68]. In all patients, NO_3_^−^ and NO_2_^−^ were measured in EBC before and after antioxidant therapy with NAC at a dose of 600 mg per day for 3 months, taken in addition to standard therapy. A change in the nature of the relationship between the content of nitrate and nitrite anions during treatment was revealed. Prior to the course of antioxidant therapy, a nonlinear relationship was observed, and at the end of therapy, a linear relationship was registered between the levels of metabolites. Thus, the findings indicate a different role of nitrite and nitrate anions in the nitric oxide cycle [68].

Due to the role of low oxygen concentration in the activation of nitrite reductase components of the NO cycle, we studied the effect of hypoxic conditions on the cycle parameters. Interval hypoxic training (IHT) was used as a model of hypoxia. Hypoxic training is used to improve physical performance and improve the function of vital systems in extreme conditions and is also used in the treatment of various diseases. In children with mild bronchial asthma, during a three-week IHT, an increase in the total concentration of nitrates and nitrites in EBC was found compared to the initial level. At the same time, they showed an improvement in the clinical state assessed by standardized assessment scales of the therapy [69].

### 1.6. Dynamic Modeling of NO Cycle

Taking into account the huge range of physiological effects of NO, there is obviously a need for the regulation of its cycle. For this purpose, a detailed understanding of the relationships of its significant components, the consistent patterns of their change, and the possibility of the numerical prediction of their concentrations are required. In this regard, there is a need for simulation modeling of NO cycling. It will give the chance to develop much more effective algorithms for the targeted effects of therapeutic agents and predict response changes in physiological parameters. Therefore, one of the main goals of modeling is the resolution of a huge number of apparent contradictions in the accumulated experimental data on NO metabolites [70–72].

The NO cycle is a complexly regulated system in which most reactions are reversible, depending on a large number of conditions. Also, numerous loops of negative and positive feedback are present in the NO cycle. Therefore, nonlinear modeling is required for an adequate approximation of such a system [70–72]. For dynamic modeling, an accurate numerical estimate of the activity of NO-synthase and nitrite reductase systems is required, depending on the presence/level of hypoxia, the intracellular concentration of ions, the ratio of hydrophobic and hydrophilic phases, the presence/absence of inflammation, etc. Creating a dynamic model of the NO cycle will allow the evaluation of possible modulating effects on the system in order to maintain or enhance the protective and physiological effects of the components of the cycle and/or limit their damaging effects.

## 2. Conclusion

The nitric oxide cycle is a highly regulated system of key importance in the functioning of the body. The formation of nitric oxide is possible without the participation of NO-synthases. Nitrate and nitrite anions can be considered as substrates in the nitrite and nitrate reductase units of the nitric oxide cycle.

Clarifying the role of the significant components of nitrite and nitrate reductase systems in the nitric oxide cycle, the mechanism of their activation and deactivation (participation of enzymes, cofactors, homeostatic indicators, etc.) under various conditions allows detailing the principles of NO cycle control. This specification will serve as the basis for the targeted effects of therapeutic agents in a variety of pathological processes of the respiratory system associated with an imbalance of multidirectional production cascades and NO modification. In the future, to understand the interrelationships of significant components of the NO cycle, the patterns of their change, and the possibility of the numerical prediction of their concentrations, there arises a need for dynamic modeling. Describing the NO cycle and its relationship to oxidative stress will enable the development of algorithms for effective diagnosis and treatment of respiratory tract diseases.
